# Validation of PhageDx^™^*Salmonella* Assay in Raw Ground Turkey and Powdered Infant Formula: AOAC *Performance Tested Method*^SM^ 121904

**DOI:** 10.1093/jaoacint/qsab037

**Published:** 2021-03-18

**Authors:** Stephen Erickson, Jose Gil, Jessica Stach, Minh M Nguyen

**Affiliations:** 1 Laboratory Corporation of America®/MedTox, 402 County Road D West, St. Paul, MN 55112, USA; 2 Laboratory of America Corporation®/National Genetics Institute, 2440 Sepulveda Blvd., Suite 235, Los Angeles, CA 90064, USA

## Abstract

**Background:**

The PhageDx^™^*Salmonella* Assay is based on the infection of *Salmonella* spp. by specific bacteriophages and expression of a luciferase reporter gene. Results are generated in as little as 9.5 h for raw ground turkey and 18.5 h for milk-based powdered infant formula (PIF).

**Objective:**

An AOAC *Performance Tested Methods*^SM^ (PTM) study was conducted to validate the PhageDx *Salmonella* Assay for the detection of *Salmonella* in 25 g raw ground turkey and 100 g PIF test portions.

**Method:**

The performance of the PhageDx *Salmonella* Assay was compared to that of the U.S. Department of Agriculture (USDA), Food Safety and Inspection Service (FSIS) *Microbiology Laboratory Guidebook* (MLG) 4.10 for raw ground turkey and the U.S. Food and Drug Administration (FDA) *Bacteriological Analytical Manual* (BAM) Chapter 5 for PIF. Inclusivity/exclusivity, product consistency and stability, and robustness testing were conducted.

**Results:**

There was no significant difference between the 25 g raw ground turkey and 100 g PIF PhageDx *Salmonella* Assay and the USDA/FSIS MLG 4.10 and FDA/BAM Chapter 5, respectively. The reporter bacteriophages were specific for *Salmonella* and infected 108 strains in inclusivity testing. They did not infect 30 non-*Salmonella* bacteria in exclusivity testing. Robustness testing showed that the method performed well with specific deviations from the standard protocol. Consistency and stability testing demonstrated that the recombinant phage gave consistent results across three production lots and was stable when stored under appropriate conditions for at least eight months.

**Conclusions:**

The data collected in the validation study demonstrate that the PhageDx *Salmonella* Assay meets the qualifications for PTM status.

**Highlights:**

The PhageDx *Salmonella* Assay is a rapid, specific, sensitive assay capable of detecting a wide range of *Salmonella* spp. with a significantly shorter turn around time than the USDA/FSIS and FDA reference methods.

## General Information

*Salmonella* is a genus of rod-shaped Gram-negative bacteria of the family *Enterobacteriaceae*. There are two species of *Salmonella*; *S. bongori* and *S. enterica*. *S. enterica* is further divided into six subspecies that include over 2600 serotypes and is responsible for a majority of reported *Salmonella* related illnesses (3).

The U.S. Department of Agriculture (USDA) has ranked *Salmonella* as one of the costliest foodborne illnesses, resulting in an estimated $3.7 billion in medical costs each year (4). The most common symptoms of a *Salmonella* infection include diarrhea, fever, and abdominal cramps and many recover without treatment. However, some cases can be so severe that they can result in hospitalization or death. The Centers for Disease Control and Prevention estimates that *Salmonella* causes about 1.2 million illnesses, 23 000 hospitalizations, and 450 deaths in the United States every year. Contaminated food accounts for about 1 million of these illnesses. In 2019, there were several outbreaks linked to papayas, tahini, raw tuna, melon, and ground turkey (5). In addition, the World Health Organization has stated that *Salmonella* contamination in powdered infant formula (PIF) remains a problem in many parts of the world (6).

## Principle 

The PhageDx *Salmonella* Assay is based on the infection of *Salmonella* spp. by bacteriophages and replication of the infecting bacteriophages within their specific hosts. Bacteriophages demonstrate a high specificity for their bacterial host and are capable of replicating within their host quickly to high numbers. The recombinant phages used in the PhageDx *Salmonella* Assay also express a luciferase reporter during replication. The presence of *Salmonella* spp*.* is determined by incubating the lysate with the appropriate luciferase substrate and detecting emitted light in a luminometer. An absence of detected light indicates that no *Salmonella* are present in that sample. An additional advantage of this system is that only viable bacteria cells are detected as bacteriophage only replicate in living cells.

## Scope of Method

- *Target organism.—Salmonella spp.*
- *Matrix.*—Raw ground turkey and PIF (milk-based).
- *Summary of validated performance claims.*—Performance equivalent to that of the USDA, Food Safety and Inspection Service (FSIS) *Microbiology Laboratory Guidebook* (MLG) 4.10, *Isolation and Identification of Salmonella from Meat, Poultry, Pasteurized Egg, and Siluriformes (Fish) Products and Carcass and Environmental Sponges* for raw ground turkey, and the U.S. Food and Drug Administration (FDA) *Bacteriological Analytical Manual* (BAM) Chapter 5 *Salmonella* for milk-based PIF (1, 2).

## Definitions

- *Probability of detection (POD).*—The proportion of positive analytical outcomes for a qualitative method for a given matrix at a given analyte level or concentration. POD is concentration dependent. Several POD measures can be calculated: POD_R_ (reference method POD), POD_C_ (confirmed candidate method POD), POD_CP_ (candidate method presumptive result POD), and POD_CC_ (candidate method confirmation result POD).
- *Difference of probabilities of detection (dPOD).*—Difference of probabilities of detection is the difference between any two POD values. If the confidence interval of a dPOD does not contain zero, then the difference is statistically significant at the 5% level.

## Materials and Methods

### Test Kit Information

For raw ground turkey:

- *Kit name.*—PhageDx *Salmonella* Assay.
- *Cat. No.*—5009.
- *Ordering information.*—Not applicable. For internal use at Laboratory Corp. of America only.

### Test Kit Components

- *PhageDx Salmonella recombinant phage.—*Part No. 3201, 12 tubes containing 100 µL phage solution.
- *Lysis buffer.—*Part No. 3010, 12 tubes containing 150 µL lysis buffer.
- *Assay buffer.—*Part No. 3003, 12 tubes containing 500 µL assay buffer.
- *Luciferase substrate.—*Part No. 3004, 12 tubes containing 10 µL luciferase substrate.
- *96-Well break-apart plate.*—Part No. 3005, one pouch containing white break-apart plate (8 wells × 12 strips).
- *One package insert.—*Part No. 3202.

### Additional Supplies and Reagents

- *Sample bags.*—WhirlPak^®^ Sterile Filter Bags for Lab Blenders, 24 oz, Cat. No. Nasco B01348WA or similar.
- *Microfuge tubes (1.5 mL).*
- *Racks for sample bag and tubes.*
- *Buffered peptone water (BPW).*—Thermo Scientific^™^ Oxoid^™^ Cat. No. OXCM0509R.
- *Adjustable single channel pipettors (10 µL–1 mL) and appropriate sterile filtered tip*s.
- *Appropriate personal protective equipment.*—*See*https://www.cdc.gov/labs/pdf/CDC-BiosafetyMicrobiologicalBiomedicalLaboratories-2009-P.PDF.
  For confirmation (optional):
- *Dynabeads^™^ anti-Salmonella.*—ThermoFisher^™^ Cat. No. 71002.
- *DynaMag^™^-2.*—Or similar, ThermoFisher Cat. No. 12321D.
- *DRG International CHROMagar^™^ Salmonella.*—Cat. No. SA132.

### Test Kit Information

For PIF:

- *Kit name*.—PhageDx *Salmonella* Assay.
- *Cat. No.*—5011.
- *Ordering information*.—Not applicable. For internal use at Laboratory Corp. of America only.

### Test Kit Components

- *PhageDx Salmonella recombinant phage.*—Part No. 3201, 12 tubes containing 100 µL phage solution.
- *Lysis buffer.*—Part No. 3010, 12 tubes containing 150 µL lysis buffer.
- *Assay buffer.*—Part No. 3003, 12 tubes containing 500 µL assay buffer.
- *Luciferase substrate.*—Part No. 3004, 12 tubes containing 10 µL luciferase substrate.
- *96-well break-apart plate.*—Part No. 3103, one pouch containing black break-apart plate (8 wells × 12 strips).
- *One package insert.*—Part No. 3203.

### Additional Supplies and Reagents

- *Sample bags.*—Fisher Scientific Cat. No. 14955196 or similar.
- *Microfuge tubes (1.5 mL).*
- *Racks for sample bag and tubes.*
- *BPW.*—Thermo Scientific Oxoid Cat. No. OXCM0509R.
- *Adjustable single channel pipettors (10 µL–1 mL) and appropriate sterile filtered tip*s.
- *Appropriate personal protective equipment.*—*See*https://www.cdc.gov/labs/pdf/CDC-BiosafetyMicrobiologicalBiomedicalLaboratories-2009-P.PDF.

For confirmation (optional):

- *DRG International CHROMagar Salmonella.*—Cat. No. SA132.

### Apparatus

- *Homogenizer.*—Stomacher^®^ 400/Stomacher 3500 or similar.
- *Air incubators capable of 37 ± 1°C*
- *Promega GloMax^®^ 96 or Navigator luminometer.*
- *Personal computer for luminometer control and data analysis.*

### Safety Precautions

- The PhageDx *Salmonella* Assay involves the enrichment of samples which may contain human pathogenic *Salmonella* and have the potential for contamination with subsequent handling of those samples. This method should be conducted by properly trained laboratory personnel in a suitable microbiology laboratory in accordance with “Biosafety in Microbiologicaland Biomedical Laboratories”, U.S. Department of Health and Human Services (7). Care should be taken when handling the sample and reagents while performing the method.
- Materials and reagents provided in the PhageDx *Salmonella* Assay are not considered hazardous if used according to the assay method. Please review the Material Safety Data Sheet prior to performing the assay.
- Follow all relevant guidelines and laboratory protocols while performing the assay and manufacturer’s equipment instructions.

### General Preparation

- Prepare BPW media according to manufacturer’s instructions.
- Before using the reagents, flick or spin the tube to collect all of the solution at the bottom of the tube.
- Due to the short enrichment times, it is vital to maintain the temperature of the sample and BPW media used in the enrichment incubation.
- Before adding the pre-warmed BPW to the sample, confirm that the media and incubator are warmed to 37 ± 1°C (PIF) or 41 ± 1°C (raw ground turkey).
- Do not allow the pre-warmed media to cool before adding to the sample.
- Maintain the media at 37 ± 1°C or 41 ± 1°C in an incubator or water bath if preparing multiple samples.
- Prepare the Promega luminometer by following the manufacturer’s cleaning procedure and make sure there are no signal “hot spots” that will affect the sample results by reading an empty plate. All signals should be less than 20 relative light units (RLUs). Set up the luminometer to read the appropriate wells on the plate and set the signal integration to 1 second with a 180 second delay between starting the program and the signal read.

### Sample Preparation

Raw ground turkey (25 g test portion):

- Weigh 25 g of raw ground turkey and place into a filter sample bag.
- Add 75 ± 5 mL pre-warmed (41 ± 1°C) BPW to the sample.
- Homogenize sample in a Stomacher 400 or equivalent. Alternatively, mix by hand.
- Loosely close the sample bag and place in a static air incubator at 41 ± 1°C for 7–18 h using a sample rack to keep the bags separate and allow heat transfer.
- Remove the enriched samples from the incubator and mix thoroughly by hand for 15–30 s and immediately proceed to the next step after mixing is completed. If sample sits for 15 min or longer, mix sample again before proceeding to the next step.
*Note:* It is critical that the enrichment is mixed well to ensure even distribution of target analyte before taking a sample aliquot.
- Using PhageDx *Salmonella* Kit Cat. No. 5009; using a single channel pipettor and fresh sterile tip for each sample, transfer 150 µL of enriched sample to white 96-well break-apart plate taking care to avoid transferring fat and meat particles as much as possible.

For PIF (100 g test portion):

- Weigh 100 g of PIF and place into a sample bag.
- Add 300 ± 5 mL pre-warmed (37 ± 1°C) BPW to the sample.
- Homogenize sample in a Stomacher 3500 at the highest setting for 120 s (or equivalent homogenizer and setting).
- Loosely close the sample bag and place in a static air incubator at 37 ± 1°C for 16–24 h using a sample rack to keep the bags separate and allow heat transfer.
- Remove the enriched samples from the incubator and mix thoroughly by hand for at least 30 s to ensure complete mixing. *Note:* Sample must be thoroughly mixed so that analyte is distributed evenly throughout the entire sample. We recommend vigorous shaking and massaging for at least 30 s and proceeding immediately to the next step after mixing is complete. If sample sits for 15 min or longer, mix sample again before proceeding to the next step.
- Using a sterile tip/pipet, transfer 1 mL of the sample to a sterile 1.5 mL microfuge tube.
- Mix contents in microfuge tube and dilute sample 1:10 in BPW (100 µL sample in 900 µL BPW) into a fresh sterile 1.5 mL tube.
- Using a single channel pipettor and clean tip for each sample, transfer 150 µL of diluted sample to black 96-well break-apart plate.

### For Both Matrixes

- After transferring samples to 96-well plates, using a single channel pipettor and clean tip for each sample, add 10 µL of the phage solution to the sample and gently mix by pipetting up and down.
- Cover plate with sealing tape to prevent cross contamination and evaporation. Place the sample in the 37 ± 1°C incubator for 2 h.
- Remove one tube containing the lysis buffer, assay buffer, and substrate for each eight well strip used and thaw to room temperature. Flick or spin the tubes to collect all of the solution at the bottom of the tubes.
- Prepare the lysis/luciferase master mix by transferring the entire contents of assay buffer (0.5 mL) and lysis buffer (150 µL) tubes to the substrate tube (10 µL) and mix well.

*Note:* Use within 1 h of preparation

**(e)** Add 65 µL of the lysis/luciferase master mix to each well using a single channel pipettor. Mix thoroughly by pipetting up and down. To avoid cross-contamination, use a clean tip for each sample.
**(f)** Once all of the samples have received the lysis/luciferase master mix, place the sample plate in the luminometer, close the lid, and initiate the read program.

### Interpretation and Test Result Report

- The luminometer program will display the results on the screen as RLU values corresponding to the well positions of the break-apart plate.
- For raw ground turkey, samples positive for *Salmonella* will have a reading value of 750 RLU or greater for a 7–13 h enrichment or 50 000 RLU or greater for >13–18 h enrichment. Negative samples will be less than 750 RLU for a 7–13 h enrichment and less than 50 000 RLU for >13–18 h enrichment.
- For PIF, samples positive for *Salmonella* will have a reading value of 500 RLU or greater. Negative samples will be less than 500 RLU.
- Once all of the samples have been run and analyzed, remove the plate from the luminometer and follow the manufacturer’s instructions for cleaning the instrument and shut down.

*Note:* In some cases,the PhageDx *Salmonella* Assay will generate a very high signal and result in adjacent wells reading as false positives due to the bleed over from the well with a high signal. If a sample well is positive and has a signal 1000 times lower than the adjacent sample well with a higher signal, this could be a result of bleed over. In these cases, we recommend that the contents of the potential false positive well (lower RLU sample) be transferred to a new well at least a 2–3 well distance from the high signal well or to a new strip and re-read to confirm that the signal is from the sample and not a result of bleed over signal.

### Confirmation

We recommend that presumptive positives from the phage assay be confirmed.

- For raw ground turkey, confirmation for *Salmonella* can be performed on overnight enriched samples using immuno-magnetic separation (IMS) particles coated with *Salmonella* antibodies (Dynabeads anti-*Salmonella*, ThermoFisher Cat. No. 71002) to capture *Salmonella* (use according to manufacturer’s instructions), and plating onto chromogenic *Salmonella* selective plates (DRG International CHROMagar *Salmonella*, Cat. No. SA132), and allowed to incubate for an additional 24 ± 2 h at 37 ± 1°C.
- For PIF*,* confirmation of *Salmonella* spp. can be performed by streaking samples enriched for a total of 24 ± 2 h directly onto *Salmonella* chromogenic selective plates (DRG International CHROMagar *Salmonella*, Cat. No. SA132). To prepare for the confirmation, allow the samples to continue enriching for a total of 24 ± 2 h at 37 ± 1°C. Remove 50–100 µL of the overnight culture and streak onto selective agar plates and incubate plates for 24 ± 2 h at 37 ± 1°C.
- Plates with colonies that appear mauve and are 1–3 mm in diameter indicate a positive result for *Salmonella* (refer to manufacturer’s product insert for detailed description).
- Alternatively, the user may use an approved reference method confirmation protocol.

## Validation Study

This validation study was conducted under the AOAC Research Institute *Performance Tested Method(s)*^SM^ (PTM) program and the *AOAC INTERNATIONAL Methods Committee Guidelines for Validation of Microbiological Methods for Food and Environmental Surfaces, Appendix J* (8). Method developer studies were conducted in the laboratories of Laboratory Corporation of America Holdings, and included the inclusivity/exclusivity study, product consistency and stability studies, and robustness testing. The independent laboratory study was conducted by Q Laboratories, Inc., and included inclusivity studies for selected strains and matrix studies for all claim matrixes.

### Method Developer Studies

**(a)** *Inclusivity and exclusivity studies*Inclusivity strains.—(*Salmonella*) were obtained from academic, governmental, and commercially available sources. Each strain was grown overnight to stationary phase in BPW media at 37 ± 1°C. The overnight cultures were then diluted to 1000 CFU/mL in BPW. One-hundred microliters of diluted cells were used to inoculate 2 × 9.9 mL of BPW to a concentration of 10 CFU/mL. Samples were then allowed to incubate at 41 ± 1°C for 7 or 18 h. At each time point, a 150 µL sample was taken for evaluation. To evaluate each strain, cells were infected with phage solution at 37 ± 1°C for 2 h. Lysis/luciferase master mix was added, and the sample was read on the luminometer. Samples enriched for 7 h with signals >750 RLU were considered positive. Strains with <750 RLU were tested again using 18 h enriched samples. Samples enriched for 18 h with >50 000 RLU were considered positive (Table 1). Exclusivity strains were also obtained from commercially available sources and were grown to stationary phase overnight. Assays with exclusivity strains were done as with inclusivity strains except overnight cultures were assayed without dilution (Table 2).

**Table 1:** Inclusivity list: *Salmonella*

No.	Organism	Serovar	Source	Strain No.	Origin	7 h enrichment	18 h enrichment^a^
1	*S. enterica,* subsp*. salamae*	58:1, z13, z28:1,5	ATCC^b^	700151	Human urine	Positive	ND^c^
2	*S. enterica,* subsp*. salamae*	1,9,12: l, w: e, n, x	ATCC	43972	Unknown^d^	Positive	ND
3	*S. enterica,* subsp*. salamae*	47: b : 1,5	ATCC	29931	Unknown	Positive	ND
4	*S. enterica;* subsp*. salamae;* serovar Dar-es-salaam	II 1,9,12: l, w: e, n, x	ATCC	6959	Urine	Positive	ND
5	*S. enterica,* subsp*. arizonae*	Not listed^e^	ATCC	BAA-1577	Unknown	Positive	ND
6	*S. enterica,* subsp*. arizonae*	51: z_4_, z_23_:-	ATCC	13314	Unknown	Negative	Positive
7	*S. enterica,* subsp*. arizonae*	Not listed	ATCC	33952	Unknown	Positive	Positive
8	*S. enterica,* subsp*. arizonae*	Ar.7:1,2,6 18: z4, z23:-	ATCC	12323	Unknown	Positive	ND
9	*S. enterica*, subsp. *arizonae*	[8:1,7,8.] 63: z4, z32:-	ATCC	700156	poultry heart	Negative	Negative
10	*S. enterica,* subsp*. diarizonae*	35: i: z	ATCC	BAA-216	Human blood	Positive	ND
11	*S. enterica,* subsp*. diarizonae*	Not listed	ATCC	BAA-639	Human feces	Positive	ND
12	*S. enterica,* subsp*. diarizonae*	47: i: z_53_: z_57_	ATCC	12325	Unknown	Positive	ND
13	*S. enterica,* subsp. *diarizonae*	Not listed	ATCC	29934	Unknown	Positive	ND
14	*S. enterica,* subsp*. diarizonae*	Not listed	ATCC	31241	Clinical isolate	Positive	ND
15	*S. enterica,* subsp*. diarizonae*	Not listed	ATCC	BAA-1579	Unknown	Positive	ND
16	*S. enterica,* subsp*. houtenae*	Not listed	USDA^f^	51158	Unknown	Positive	ND
17	*S. enterica*, subsp. *houtenae*	45: g, z51:-	ATCC	43974	Unknown	Negative	Negative
18	*S. enterica,* subsp*. houtenae*	Not listed	ATCC	BAA-1580	Unknown	Positive	ND
19	*S. enterica,* subsp*. indica*	1,6,14,25: a: e, n, x	ATCC	43976	Unknown	Positive	ND
20	*S. enterica,* subsp*. indica*^g^	Not listed	ATCC	BAA-1578	India	Positive	Positive
21	*S. enterica,* subsp*. indica*^g^	1,6,14,25: a: e, n, x	NCTC^h^	10458	Coconut	Positive	Positive
22	*S. enterica,* subsp*. indica*^g^	Not listed	Q Labs^i^	QL 024.62	Unknown	Positive	Positive
23	*S. enterica,* subsp*. indica*^g^	Not listed	Q Labs	QL 18022.6	Unknown	Positive	Positive
24	*S. bongori*	66: z41:-	ATCC	43975	Unknown	Positive	ND
25	*S. bongori* ^g^	66: z41:-	NCTC	12419	Unknown	Positive	Positive
26	*S. bongori* ^g^	66: z41:-	NCTC	10946	Frog	Positive	Positive
27	*S. enterica,* subsp*. enterica,* serovar Adelaide	O	USDA	SEP293	Unknown	Positive	ND
28	*S. enterica,* subsp*. enterica,* serovar Abaetetuba	F	ATCC	35640	Creek water	Positive	ND
29	*S. enterica,* subsp*. enterica*, serovar Abony	B	ATCC	BAA-2162	Unknown	Positive	ND
30	*S. enterica,* subsp*. enterica,* serovar Agona	B	FDA^j^	SARB 1	Peru	Positive	ND
31	*S. enterica,* subsp*. enterica,* serovar Alachua	O	University of Iowa^k^	DMS012	Unknown	Positive	ND
32	*S. enterica,* subsp*. enterica,* serovar Amsterdam	E_1_	USDA	41084	Unknown	Positive	ND
33	*S. enterica,* subsp*. enterica*, serovar Anatum	E_1_	FDA	SARB 2	Human, WA	Positive	ND
34	*S. enterica,* subsp. *enterica,* serovar Bareilly	C_1_	University of Georgia^l^	73	Unknown	Positive	ND
35	*S. enterica,* subsp*. enterica,* serovar Benfica	E_1_	USDA	AUG071	Unknown	Positive	ND
36	*S. enterica,* subsp. *enterica,* serovar Bispebjerg	B	ATCC	9842	Unknown	Positive	ND
37	*S. enterica,* subsp*. enterica,* serovar Brandenburg	B	USDA	AUG053	Unknown	Positive	ND
38	*S. enterica,* subsp*. enterica*, serovar Braenderup	C_1_	USDA	52115	Unknown	Positive	ND
39	*S. enterica,* subsp*. enterica*, serovar Bredeney	B	USDA	61003.2	Unknown	Positive	ND
40	*S. enterica*, subsp*. enterica,* serovar Breukelan	C_2_	ATCC	15782	Cuscus	Positive	ND
41	*S. enterica,* subsp*. enterica,* serovar Cerro	K	USDA	31011.1	Unknown	Positive	ND
42	*S. enterica,* subsp*. enterica,* serovar Champaign	Q	ATCC	700139	Hen liver	Positive	ND
43	*S. enterica,* subsp*. enterica,* serovar Chester	B	ATCC	11997	Unknown	Positive	ND
44	*S. enterica*, subsp*. enterica*, serovar Choleraesuis	6,7: c; 1,5	ATCC	10708	Unknown	Positive	ND
45	*S. enterica,* subsp*. enterica,* serovar Choleraesuis A	6,7: c; 1,5	ATCC	7001	Unknown	Positive	ND
46	*S. enterica,* subsp*. enterica*, serovar Derby	B	FDA	SARB 11	Turkey, PA	Positive	ND
47	*S. enterica,* subsp. *enterica,* serovar Dublin	D_1_	FDA	SL477	Unknown	Positive	ND
48	*S. enterica,* subsp*. enterica,* serovar Eko	B	USDA	33006.2	Unknown	Positive	ND
49	*S. enterica,* subsp*. enterica,* serovar Enteritidis	D_1_	FDA	SARB 17	Brazil	Positive	ND
50	*S. enterica*, subsp*. enterica,* serovar Gallinarum	D_1_	University of Iowa	4-50-39	Unknown	Positive	ND
51	*S. enterica,* subsp*. enterica,* serovar Hadar	C_2_	University of Georgia	MH44684	Swine	Positive	ND
52	*S. enterica,* subsp. *enterica,* serovar Havana	G	University of Georgia	MH84665	Unknown	Positive	ND
53	*S. enterica,* subsp*. enterica,* serovar Heidelberg	B	FDA	SL476	Unknown	Positive	ND
54	*S. enterica,* subsp*. enterica,* serovar Hvittingfoss	I	USDA	63008.2	Unknown	Positive	ND
55	*S. enterica,* subsp. *enterica, serovar* Illinois	E_1_	ATCC	11646	Unknown	Positive	ND
56	*S. enterica,* subsp. *enterica,* serovar Infantis	C_1_	University of Georgia	MH95276	Unknown	Positive	ND
57	*S. enterica,* subsp*. enterica,* serovar Javiana	D_1_	ATCC	10721	Unknown	Positive	ND
58	*S. enterica,* subsp*. enterica,* serovar Kahla	T	ATCC	17980	Feces	Positive	ND
59	*S. enterica,* subsp*. enterica,* serovar Kalamu	B	USDA	63279.2	Unknown	Positive	ND
60	*S. enterica,* subsp*. enterica,* serovar Kentucky	C_2_	ATCC	9263	Unknown	Positive	ND
61	*S. enterica,* subsp. *enterica,* serovar Kiambu	B	USDA	51316	Unknown	Positive	ND
62	*S. enterica,* subsp*. enterica,* serovar Lexington	E_1_	University of Georgia	9492-M	Unknown	Positive	ND
63	*S. enterica*, subsp*. enterica,* serovar Liverpool	E_4_	USDA	AUG365	Unknown	Positive	ND
64	*S. enterica,* subsp*. enterica,* serovar London	E_1_	USDA	JUL218	Unknown	Positive	ND
65	*S. enterica,* subsp*. enterica,* serovar Mbandaka	C_1_	University of Georgia	74	Unknown	Positive	ND
66	*S. enterica*, subsp*. enterica,* serovar Meleagridis	E_1_	USDA	FEB095	Unknown	Positive	ND
67	*S. enterica,* subsp. *enterica,* serovar Menden	C_1_	ATCC	15992	Feces	Positive	ND
68	*S. enterica,* subsp*. enterica,* serovar Minnesota	L	USDA	52329.1	Unknown	Positive	ND
69	*S. enterica,* subsp*. enterica,* serovar Michigan	J	University of Georgia		Unknown	Positive	ND
70	*S. enterica,* subsp*. enterica,* serovar Mississippi	G	University of Iowa	DMSO49	Unknown	Positive	ND
71	*S. enterica,* subsp. *enterica,* serovar Monophasic	Not listed	University of Georgia	102	Unknown	Positive	ND
72	*S. enterica, subsp. enterica,* serovar Montevideo	C_1_	ATCC	8387	Unknown	Positive	ND
73	*S. enterica,* subsp*. enterica,* serovar Muenchen	C_2_	FDA	SARB 35	Human, NC	Positive	ND
74	*S. enterica,* subsp*. enterica,* serovar Muenster	E_1_	USDA	31053	Unknown	Positive	ND
75	*S. enterica,* subsp*. enterica,* serovar Newport	C_2_	FDA	SL317	Unknown	Positive	ND
76	*S. enterica,* subsp*. enterica,* serovar Ngili	C_1_	ATCC	19127	Feces	Positive	ND
77	*S. enterica,* subsp. *enterica,* serovar Ohio	C_1_	USDA	52307	Unknown	Positive	ND
78	*S. enterica,* subsp*. enterica,* serovar Oranienburg	C_1_	ATCC	9239	Unknown	Positive	ND
79	*S. enterica,* subsp*. enterica,* serovar Panama	D_1_	FDA	SARB 40	Human, NC	Positive	ND
80	*S. enterica,* subsp*. enterica,* serovar Paratyphi A	A	ATCC	9150	Unknown	Positive	ND
81	*S. enterica,* subsp*. enterica,* serovar Paratyphi B	B	USDA	SEP358	Unknown	Positive	ND
82	*S. enterica,* subsp*. enterica,* serovar Paratyphi C	C_1_	ATCC	BAA-1714	Unknown	Positive	ND
83	*S. enterica,* subsp. *enterica,* serovar Pomona	M	University of Iowa	DMSO63	Unknown	Positive	ND
84	*S. enterica,* subsp*. enterica,* serovar Potsdam	C_1_	ATCC	25957	Child	Positive	ND
85	*S. enterica,* subsp*. enterica,* serovar Pullorum	D_1_	ATCC	13036	Egg	Positive	ND
86	*S. enterica*, subsp*. enterica,* serovar Reading	B	USDA	SEP245	Unknown	Positive	ND
87	*S. enterica,* subsp*. enterica,* serovar Remo	B	USDA	43164.2	Unknown	Positive	ND
88	*S. enterica,* subsp. *enterica,* serovar Rubislaw	F	University of Iowa	DMSO67	Unknown	Positive	ND
89	*S. enterica,* subsp*. enterica,* serovar Saintpaul	B	ATCC	9712	Cystitis	Positive	ND
90	*S. enterica,* subsp*. enterica,* serovar San Diego	B	USDA	APR025	Unknown	Positive	ND
91	*S. enterica,* subsp*. enterica,* serovar Schwarzengrund	B	USDA	13092.2	Unknown	Positive	ND
92	*S. enterica,* subsp*. enterica,* serovar Senftenburg	E_4_	FDA	SARB 59	Chicken, MA	Positive	ND
93	*S. enterica,* subsp. *enterica,* serovar Simsbury	E_4_	ATCC	12004	Unknown	Positive	ND
94	*S. enterica,* subsp*. enterica,* serovar Stanley	B	ATCC	7308	Unknown	Positive	ND
95	*S. enterica,* subsp. *enterica,* serovar Taksony	E_4_	USDA	32133	Unknown	Positive	ND
96	*S. enterica,* subsp*. enterica,* serovar Tallahassee	C_2_	ATCC	12002	Unknown	Positive	ND
97	*S. enterica,* subsp. *enterica,* serovar Tennessee	C_1_	FDA	SL487	Peanut butter	Positive	ND
98	*S. enterica,* subsp*. enterica,* serovar Thompson	C_1_	University of Georgia	11842M	Horse	Positive	ND
99	*S. enterica,* subsp*. enterica,* serovar Typhi C	C_1_	ATCC	BAA-6539	Unknown	Positive	ND
100	*S. enterica,* subsp*. enterica,* serovar Typhimurium	B	FDA	1226	Unknown	Positive	ND
101	*S. enterica, subsp. enterica, serovar* Typhimurium DT104	B	FDA	1294	Outbreak set	Positive	ND
102	*S. enterica,* subsp*. enterica,* serovar Typhimurium/DT104b	B	FDA	1278	Outbreak set	Positive	ND
103	*S. enterica,* subsp*. enterica,* serovar Uganda	E_4_	USDA	51278.2	Unknown	Positive	ND
104	*S. enterica,* subsp. *enterica,* serovar Urbana	N	ATCC	9261	Unknown	Positive	ND
105	*S. enterica,* subsp*. enterica,* serovar Vellore	B	ATCC	15611	Rectal swab	Positive	ND
106	*S. enterica*, subsp*. enterica,* serovar Virchow	C_1_	ATCC	51955	Unknown	Positive	ND
107	*S. enterica,* subsp. *enterica,* serovar Wagadugu	E_1_	USDA	53298	Unknown	Positive	ND
108	*S. enterica,* subsp. *enterica,* serovar Weltevreden	E_1_	ATCC	BAA-2568	Unknown	Positive	ND
109	*S. enterica,* subsp. *enterica,* serovar Worthington	G_2_	ATCC	BAA-2085	Unknown	Positive	ND
110	*Salmonella* non-typeable	Not listed	USDA	63214	Unknown	Positive	ND

a 18 h enrichments were not tested if 7 h enrichments were positive based on the assumption that at 18 h there would be a greater number of cells and thus would also result in a positive result.

b American Type Culture Collection, Manassas, VA.

c ND = Not done.

d Unknown = No information is available on the origin of the strain.

e Serovar or antigenic formula not listed for this strain by the source.

f U.S. Department of Agriculture, Animal Research Center, Clay Center, NE.

g Inclusivity assay performed by Q Laboratories.

h National Collection of Type Cultures, Porton Down, Salisbury, UK.

I Q Laboratories, Cincinnati, OH.

j U.S. Food and Drug Administration, Center for Food Safety and Applied Nutrition, College Park, MD.

k University of Iowa, Iowa City, IA.

l University of Georgia, Athens, GA.

**Table 2.** Exclusivity list

No.	Organism	Source	Strain ID	Origin	PhageDx result
1	*Acinetobacter baumannii*	ATCC^a^	19606	Urine	Negative
2	*Bacillus cereus*	ATCC	14579	Unknown^b^	Negative
3	*B.* *subtilis subsp. subtilis*	ATCC	6051	Unknown	Negative
4	*Citrobacter* *freundii*	ATCC	8090	Unknown	Negative
5	*C.* *werkmanii*	ATCC	51114	Human blood	Negative
6	*C.* *youngae*	ATCC	29935	Metal scraps	Negative
7	*C.* *koseri*	ATCC	25408	Throat	Negative
8	*Cronobacter sakazakii*	ATCC	BAA-894	Human clinical	Negative
9	*Escherichia coli*	ATCC	25922	Clinical	Negative
10	*E.* *coli 0157: H7 (stx-)*	ATCC	43888	Human feces	Negative
11	*Edwardsiella tarda*	ATCC	15947	Stool	Negative
12	*Enterobacter cloacae subsp cloacae*	ATCC	13047	Spinal Fluid	Negative
13	*E.* *kobei*	ATCC	BAA-260	Human blood	Negative
14	*Enteroccus faecium*	ATCC	19434	Unknown	Negative
15	*E.* *faecalis*	ATCC	29212	Urine	Negative
16	*Escherichia fergusonii*	ATCC	35469	Human feces	Negative
17	*E.* *hermanni*	ATCC	33650	Clinical, toe	Negative
18	*Hafnia alevi*	ATCC	13337	Unknown	Negative
19	*Klebsiella oxytoca*	ATCC	43165	Clinical	Negative
20	*K.* *pneumoniae*	ATCC	4352	Cow's milk	Negative
21	*Listeria grayi*	ATCC	25401	Corn stalks, leaves	Negative
22	*L.* *welshimeri*	ATCC	35897	Decaying plant material	Negative
23	*Morganella morganii: subsp. Maorganii M11*	ATCC	25830	Clinical	Negative
24	*Pluralibacter gergoviae*	ATCC	33028	Urine	Negative
25	*Proteus mirabilis*	ATCC	43071	Clinical, toe	Negative
26	*Pseudomonas aeruginosa; Strain Boston 41401*	ATCC	27853	Blood culture	Negative
27	*Shigella sonnei*	ATCC	9290	Unknown	Negative
28	*Staphylococcus aureus*	ATCC	29213	Wound	Negative
29	*S.* *epidermidis*	ATCC	14990	Nose	Negative
30	*Yersinia enterocolitica*	ATCC	23715	Human blood	Negative

a American Type Culture Collection, Manassas, VA.

b Unknown = No information is available on the origin of the strain.

**(b)** *Product consistency (lot-to-lot) and stability studies*.—Three separate production lots of PhageDx *Salmonella* recombinant phage were prepared according to written manufacturing documents and tested according to quality control procedures. Quality control procedures verified that each lot when diluted to working concentration had the similar titer, background, and level of detection (LOD). Recombinant phage reagents were aged between 1 and 6 months when assayed for stability.

Consistency and stability were done according to AOAC guidance, where a sample was inoculated with *S. typhimurium*, American Type Culture Collection (ATCC) 19585, to give fractional positives. Ten replicates were run in the PhageDx Assay, and the RLU values analyzed. A set of stability studies was also conducted using the non-target bacterium *Citrobacter freundii* (ATCC 8090)*.* Overnight cultures of *C. freundii* were used directly in the assay. Results are shown in Table 3.

**Table 3.** Stability and consistency (lot-to-lot) of PhageDx *Salmonella* recombinant phage—POD comparison

Phage lot No.	Lot age, months	N^a^	x^b^	POD_A_^c^	95% CI	Phage lot No.	Lot age, months	N	x	POD_B_^d^	95% CI	dPOD_AB_^e^	95% CI^f^
*S.* Typhimurium (target)													
B^h^	3	10	6	0.6	0.31, 0.83	C^i^	1	10	6	0.6	0.31, 0.83	0.00	−0.37, 0.37
A^g^	8	10	4	0.4	0.17, 0.69	C	1	10	6	0.6	0.31, 0.83	−0.20	−0.53, 0.21
A	8	10	4	0.4	0.17, 0.69	B	3	10	6	0.6	0.31, 0.83	−0.20	−0.53, 0.21
*Citrobacter freundii* (non-target)													
B	3	10	0	0.0	0.0, 0.28	C	1	10	0	0.0	0.0, 0.28	0.0	−0.28, 0.28
A	8	10	0	0.0	0.0, 0.28	C	1	10	0	0.0	0.0, 0.28	0.0	−0.28, 0.28
A	8	10	0	0.0	0.0, 0.28	B	3	10	0	0.0	0.0, 0.28	0.0	−0.28, 0.28

a N = Number of test portions.

b x = Number of positive test portions.

c POD_A_ = Positive outcomes divided by the total number of trials first member of pair.

d POD_B_ = Positive outcomes divided by the total number of trials second member of pair.

e dPOD_AB_ = Difference in POD between the paired comparison.

f 95% CI = If the confidence interval of a dPOD does not contain zero, then the difference is statistically significant at the 5% level.

g Lot Awas produced 12/18.

h Lot B was produced 03/19.

i Lot C was produced 08/19.

**(c)** *Robustness study*.—Three parameters were varied to demonstrate assay robustness: enrichment time (6.5 and 24 h), recombinant phage concentration (±20%), and lysis/luciferase master mix amount (±5 µL). Briefly, 25 g raw ground turkey samples were left unspiked or spiked with 0.2–2 CFU/25 g with *S. Heidelberg* SL476 and stored at 2–8°C for 48–72 h. The PhageDx *Salmonella* Assay protocol was followed with the variations in enrichment time, recombinant phage concentration, and lysis/substrate master mix amounts as indicated in Table 4. Samples with RLU values greater than 750 were considered positive for 6.5 and 7 h enriched samples and RLU values greater than 50 000 were considered positive for the 24 h enriched samples. Samples were confirmed by allowing samples to enrich overnight and performing IMS with anti-*Salmonella* coated particles and plating on chromogenic *Salmonella* selective plates. The presence of mauve colonies that are 1–3 mm in diameter on selective plates indicate a positive result for *Salmonella*. A summary of the testing is presented in Table 4.

**Table 4.** Robustness study: impact of varying enrichment time, phage concentration, lysis/luciferase master mix concentration on PhageDx *Salmonella* Assay results—POD comparison

Test condition^a^	Test parameters			N^c^	Test condition results			Nominal condition results^b^			dPOD_TN_^g^	95% CI^h^
	Enrichment time, h	Volume phage, µL	Volume substrate		x^d^	${\text{POD}}_{T}^{e}$	95% CI	x	POD_N_^f^	95% CI		
Raw ground turkey—spiked with *S.* Heidelberg (target)												
1	6.5	8	60	10	7	0.7	0.40, 0.89	7	0.7	0.40, 0.89	0.0	−0.25, 0.25
2	6.5	8	70	10	7	0.7	0.40, 0.89	7	0.7	0.40, 0.89	0.0	−0.25, 0.25
3	6.5	12	60	10	7	0.7	0.40, 0.89	7	0.7	0.40, 0.89	0.0	−0.25, 0.25
4	6.5	12	70	10	7	0.7	0.40, 0.89	7	0.7	0.40, 0.89	0.0	−0.25, 0.25
5	24	8	60	10	7	0.7	0.40, 0.89	7	0.7	0.40, 0.89	0.0	−0.25, 0.25
6	24	8	70	10	7	0.7	0.40, 0.89	7	0.7	0.40, 0.89	0.0	−0.25, 0.25
7	24	12	60	10	7	0.7	0.40, 0.89	7	0.7	0.40, 0.89	0.0	−0.25, 0.25
8	24	12	70	10	7	0.7	0.40, 0.89	7	0.7	0.40, 0.89	0.0	−0.25, 0.25
Raw ground turkey—unspiked (non-target)												
1	6.5	8	60	10	0	0.0	0.00, 0.28	0	0.0	0.00, 0.28	0.0	−0.25, 0.25
2	6.5	8	70	10	0	0.0	0.00, 0.28	0	0.0	0.00, 0.28	0.0	−0.25, 0.25
3	6.5	12	60	10	0	0.0	0.00, 0.28	0	0.0	0.00, 0.28	0.0	−0.25, 0.25
4	6.5	12	70	10	0	0.0	0.00, 0.28	0	0.0	0.00, 0.28	0.0	−0.25, 0.25
5	24	8	60	10	0	0.0	0.00, 0.28	0	0.0	0.00, 0.28	0.0	−0.25, 0.25
6	24	8	70	10	0	0.0	0.00, 0.28	0	0.0	0.00, 0.28	0.0	−0.25, 0.25
7	24	12	60	10	0	0.0	0.00, 0.28	0	0.0	0.00, 0.28	0.0	−0.25, 0.25
8	24	12	70	10	0	0.0	0.00, 0.28	0	0.0	0.00, 0.28	0.0	−0.25, 0.25

a Each test condition is being compared to the nominal test condition.

b Nominal condition = 7 h enrichment, 10 µL phage, 65 µL lysis/luciferase master mix substrate.

c N = Number of test portions per condition.

d x = Number of positive test portions per condition.

e POD_T_ = Positive outcomes divided by the total number of trials per condition.

f POD_N_ = Positive outcomes divided by the total number of trials per nominal condition.

g dPOD_TN_ = Difference in POD between the test condition and nominal condition.

h 95% CI = If the confidence interval of a dPOD does not contain zero, then the difference is statistically significant at the 5% level.

### Independent Laboratory Validation Study

**(a)** *Inclusivity*.—For the inclusivity study six strains of *Salmonella* were evaluated. Each *Salmonella* strain evaluated was cultured by transferring a single colony from trypticase soy agar with 5% sheep blood (SBA) to a 9 mL aliquot of BPW for 7 h at 41 ± 1°C, and to a second 9 mL aliquot of BPW for 16 h at 37 ± 1°C. After incubation each *Salmonella* strain at each culture condition was then diluted to 100× the LOD of the PhageDx *Salmonella* Assay and analyzed. Tests results were reported as either positive or negative (Table 1).

####  

**(b)** *Matrix study*.—The independent laboratory evaluation included matrix studies for raw ground turkey and milk-based PIF comparing the PhageDx *Salmonella* Assay to USDA/FSIS MLG 4.10 and FDA/BAM Chapter 5, respectively. Within each sample set, there were five uninoculated samples (0 CFU/test portion), 20 low-level inoculated samples (0.2–2 CFU/test portion), and five high-level inoculated samples (2–10 CFU/test portion). The low inoculation level was designed to produce fractional positive results in which the candidate or reference method produced 5–15 positive results (25–75%).

The raw ground turkey and milk-based PIF were purchased from a local supplier and prescreened for natural contamination of the analyte following USDA/FSIS MLG 4.10 and the FDA/BAM Chapter 5 reference methods, respectively. Total aerobic count was determined following FDA/BAM Chapter 3 *Aerobic Plate Count* reference method (9). Following the screening, the matrixes were inoculated with the indicated strains of *Salmonella* species. For raw ground turkey matrix, a liquid inoculum culture was used. The inoculum was prepared by transferring a single *Salmonella* colony from a stock culture stored at –70°C on SBA into brain heart infusion (BHI) broth and incubating the culture at 35 ± 1°C for 24 ± 2 h. Following incubation, the culture was diluted to a target level using BHI as the diluent to a low level expected to yield fractional positive results (5–15 positive results), and a high level expected to yield all positive results. Samples were spiked and held for 48–72 h post-inoculation at 2–8°C to allow for equilibration of the organism as per AOAC Guidelines.

For the milk-based PIF matrix a lyophilized culture was used. *Salmonella* were cultured from stock stored at –70°C on SBA for 18 hr at 37°C. The lyophyilized culture was prepared by inoculating BHI broth with a single colony from SBA and incubating for 18–24 h at 35 ± 2°C, diluting the culture into a sterile cryoprotectant, adding non-fat dried milk (NFDM), and freeze dried for 48–72 h. The culture was then diluted in a sterile cryoprotectant, reconstituted NFDM, and freeze dried for 48–72 h. A bulk lot of the matrix was inoculated with a lyophilized culture that was diluted in powdered NFDM to a low level expected to yield fractional positive results (5–15 positive results), and a high level expected to yield all positive results. After inoculation, samples were held for 2 weeks at room temperature (24 ± 2°C) to allow for equilibration of the organism as per AOAC guidelines. For all 100 g test portions analyzed, 25 g of inoculated matrix at each level of contamination was transferred to sterile filter laboratory blender bags on the day of analysis, and then 75 g of uninoculated matrix added to create 100 g test portions.

The level of *Salmonella* in the low-level inoculum and high-level inoculum was determined by most probable number (MPN) on the day of analysis. For the 25 g test portion samples, low-level inoculum MPN was determined by evaluating 5 × 50 g, 20 × 25 g reference method test portions from the study, and 5 × 10 g inoculated test portions. The level of *Salmonella* in the high-level inoculum in 25 g test portions was determined by evaluating the 5 × 25 g reference method test portions from the study, 5 × 10 g, and 5 × 5 g inoculated test portions. To the 50 g portions, 450 mL of the reference method enrichment broth was added; to the 10 g portions, 90 mL of the reference method enrichment broth was added; and to the 5 g portions, 45 mL enrichment broth was added. All 25 g portions were utilized from reference method test potions and analyzed following the FDA/BAM Chapter 5 reference method. The number of positives from the three test levels was used to calculate the MPN using the LCF MPN calculator (version 1.6) (10).

### PhageDx Salmonella assay

All samples were analyzed by the PhageDx *Salmonella* Assay following enrichment with pre-warmed (41 ± 1°C) BPW and incubated for 7 and 18 h at 41 ± 1°C for raw ground turkey, and enrichment with pre-warmed (37 ± 1°C) BPW and incubated for 16 and 24 h at 37 ± 1°C for milk-based PIF. After enrichment, a 150 µL direct sample for raw ground turkey, or a 150 µL 1:10 diluted sample for PIF, was transferred to a 96-well plate. Ten microliters of the phage reagent were added and samples were incubated at 37 ± 1°C for 2 h. Then, 65 µL of lysis/luciferase master mix was added and the samples read on a luminometer. Regardless of presumptive results, all samples were culturally confirmed by the USDA/FSIS MLG 4.10 or FDA/BAM Chapter 5 reference method. In addition, all samples were confirmed following the alternative confirmation described in *Sample Preparation*, subsection *Confirmation*. Final confirmation for all samples was obtained by Bruker MALDI Biotyper following AOAC Method **2017.09** (11).

### USDA/FSIS MLG 4.10

For the USDA/FSIS MLG 4.10, 25 ± 2.5 g of raw ground turkey portions were combined with 225 ± 4.5 mL of BPW, homogenized by stomaching for 2 min and incubated 18–24 h at 35 ± 2°C. After incubation of all test portions, 0.5 ± 0.05 mL of the sample enrichment was transferred into 10 ± 0.5 mL of tetrathionate (TT) broth Hajna, and 0.1 ± 0.02 mL of the sample enrichment was transferred into 10 ± 0.5 mL of modified Rappaport Vassiliadis (mRV) medium. The secondary enrichments were incubated in a circulating, thermostatic water bath at 42 ± 0.5°C for 18–24 h.

After 18–24 h, the contents in the TT and mRV enrichments were mixed by vortex and a loopful of each streaked to xylose lysine tergitol 4 (XLT4) agar and brilliant green sulfa agar (BGSA). All plates were incubated at 35 ± 2° C for 18–24 h. After incubation, plates were observed for typical and well-isolated colonies. One typical colony for each positive sample was picked to triple sugar iron (TSI) agar and lysine iron agar (LIA) slants, along with tryptic soy agar (TSA) plates, and incubated for 24 ± 2 h at 35 ± 2°C. Following incubation, the slants were examined as a set and the biochemical reactions of the slants noted. Final confirmation was obtained from purified TSA isolates using the Bruker MALDI Biotyper following AOAC Method **2017.09**.

### FDA/BAM Chapter 5 *Salmonella*

For the FDA/BAM reference method, 25 g milk-based PIF portions were combined with 225 ± 5 mL of lactose broth and homogenized by stomaching for 2 min. Following homogenization, test portions were allowed to stand at room temperature (24 ± 2°C) for 60 ± 5 min. If necessary, the pH of the enrichments for all matrices was adjusted to 6.8 ± 0.2. Subsequently, all matrix enrichments were incubated at 35 ± 2°C for 24 ± 2 h.

Following incubation, 0.1 mL of primary enrichment was transferred into 10 mL of RV and 1.0 mL into 10 mL of TT medium. RV tubes were incubated at 42 ± 0.2°C for 24 ± 2 h. The milk-based PIF tested had a low microbial background (<10^4^ CFU/g); therefore, the TT tubes were incubated at 35 ± 2°C for 24 ± 2 h. Following incubation, a loopful of the secondary enrichments were streaked to bismuth sulfite (BS), Hektoen enteric (HE) and xylose lysine deoxycholate (XLD) agar and incubated at 35 ± 2°C for 24 ± 2 h. If no visible colonies were present after 24 h of incubation on the BS plates, they were re-incubated for an additional 24 ± 2 h at 35 ± 2°C. A minimum of two suspect colonies from each selective agar were transferred to TSI and LIA slants and incubated at 35 ± 2°C for 24 ± 2 h. Following incubation, TSI and LIA slants were examined for typical reactions. Slants producing typical reactions were streaked to TSA and incubated for 35 ± 2°C for 18–24 h.

Following incubation, isolates were serologically tested for both somatic O and flagellar H agglutination. Additionally, final confirmation was obtained from purified TSA isolates using the Bruker MALDI Biotyper following AOAC Method **2017.09**.

### Results

Inclusivity and exclusivity studies using the PhageDx *Salmonella* Assay demonstrate that the PhageDx Assay is specific for the detection of *Salmonella* spp. The PhageDx *Salmonella* Assay was able to detect 108/110 *Salmonella* strains tested (Table 1). In addition, the PhageDx Assay did not detect 30/30 non-*Salmonella* strains tested (Table 2).

Product consistency (lot-to-lot) and stability studies show that the PhageDx *Salmonella* recombinant phages can be manufactured consistently and are stable for at least 8 months when stored at 4°C. Manufactured lots were made on 12/18, 3/19, and 8/19 according to written manufacturing documents. Working solutions of each lot produced similar results when tested according to QC tests for bacteriophage concentration, background signal, and LOD. Stability tests of each lot were performed to determine the shelf life of the recombinant phage. These tests demonstrated that lots produced 1 month prior to testing showed no significant difference from lots produced at least 8 months prior to testing. Additionally, no variation in exclusivity was observed with these three recombinant phage lots in tests with *C. freundii*.

Robustness testing of the PhageDx *Salmonella* Assay demonstrated that variations in enrichment time, recombinant phage concentration, and lysis/luciferase master mix amounts do not alter the results compared to the standard protocol. Enrichment times of 6.5 and 24 h, recombinant phage volumes of 8 and 12 μL, and lysis/luciferase master mix volumes of 60 and 70 μL produced identical results to the standard protocol of 7 h enrichment, 10 μL of recombinant phage, and 65 μL of lysis/luciferase master mix in both uninoculated and low inoculum test samples (Table 4). These results indicate that these deviations from the PhageDx *Salmonella* Assay protocol did not alter the final results.

In an unpaired study, the presumptive results from the PhageDx *Salmonella* Assay for raw ground turkey (7 and 18 h enrichments) and PIF (16 and 24 h enrichments) were not significantly different from those of the USDA/FSIS MLG 4.10 and FDA/BAM Chapter 5, respectively. In a paired study, the results from the PhageDx *Salmonella* Assay presumptive, PhageDx confirmation method, and the respective reference methods were identical (Table 6). In addition, no false positive or false negatives were detected in the matrix study. In summary, independent laboratory matrix tests demonstrated that the results from PhageDx *Salmonella* Assay and the USDA/FSIS MLG 4.10 and FDA/BAM chapter 5 reference methods for raw ground turkey and PIF, respectively, were not significantly different (Tables 5 and 6).

**Table 5.** PhageDx *Salmonella* Assay results versus reference method comparison results

Matrix^a^	Strain	Enrichment time point^b^	MPN/test portion^c^	N^d^	PhageDx *Salmonella* result			Reference method result			dPOD_CP_^h^	95% CI^i^
					X^e^	POD_CP_^f^	95% CI	x	POD_CC_^g^	95% CI		
Raw ground turkey (25 g)^j^	*S.* Enteritidis ATCC 13076^k^		N/A^l^	5	0	0.00	0.00, 0.43	0	0.00	0.00, 0.43	0.00	−0.43, 0.43
		7 and 18 h	0.55 (0.29, 093)	20	7	0.35	0.18, 0.57	8	0.40	0.22, 0.61	−0.05	−0.32, 0.23
			1.74 (0.77, 4.03)	5	5	1.00	0.57, 1.00	5	1.00	0.57, 1.00	0.00	−0.43, 0.43
PIF milk- based (100g)^j^	*S.* Typhimurium ATCC BAA-215		N/A	5	0	0.00	0.00, 0.43	0	0.00	0.00, 0.43	0.00	−0.43, 0.43
		16 and 24 h	0.68 (0.39, 1.12)	20	9	0.45	0.26, 0.66	8	0.40	0.22, 0.61	0.05	−0.24, 0.33
			3.70 (1.52, 9.02)	5	5	1.00	0.57, 1.00	5	1.00	0.57, 1.00	0.00	−0.43, 0.43

a Matrix study was unpaired and analyzed by the unpaired POD statistical analysis.

b Both enrichment time points produced identical results.

c MPN is based on the POD of reference method test portions using the Least Cost Formulations MPN calculator, with 95% confidence interval.

d N = Number of test portions.

e x = Number of positive test portions.

f POD_CP_ = Candidate method presumptive positive outcomes confirmed positive.

g POD_CC_ = Reference method confirmed positive outcomes divided by the total number of trials.

h dPOD_CP_ = Difference between the candidate method and reference method POD values.

i 95% CI = If the confidence interval of a dPOD does not contain zero, then the difference is statistically significant at the 5% level.

j Matrix tested by the independent laboratory.

k ATCC = American Type Culture Collection, Manassas, VA.

l N/A = Not applicable.

**Table 6.** PhageDx *Salmonella* Assay presumptive versus confirmed—POD result

Matrix	Strain	Enrichment time points^a^	MPN/test portion^b^	N^c^	Presumptive			Confirmed^d^			${\text{dPOD}}_{\text{CP}}^{h}$	95% CI^i^
					X^e^	${\text{POD}}_{\text{CP}}^{f}$	95% CI	X	${\text{POD}}_{\text{CC}}^{g}$	95% CI		
Raw ground turkey (25 g)^j^	*S.* Enteritidis ATCC 13076^k^	7 and 18 h	N/A^l^	5	0	0.00	0.00, 0.43	0	0.00	0.00, 0.43	0.00	−0.47, 0.47
			0.55 (0.29, 0.93)	20	7	0.35	0.18, 0.57	7	0.35	0.18, 0.57	0.00	−0.28, 0.28
			2.76 (1.51, 5.78)	5	5	1.00	0.57, 1.00	5	1.00	0.57, 1.00	0.00	−0.47, 0.47
PIF milk- based (100g)^j^	*S.* Typhimurium ATCC BAA-215	16 and 24 h	N/A	5	0	0.00	0.00, 0.43	0	0.00	0.00, 0.43	0.00	−0.47, 0.47
			0.68 (0.39, 1.12)	20	9	0.45	0.26, 0.66	9	0.45	0.26, 0.66	0.00	−0.28, 0.28
			3.70 (1.52, 9.02)	5	5	1.00	0.57, 1.00	5	1.00	0.57, 1.00	0.00	−0.47, 0.47

a Both enrichment time points produced identical results.

b MPN is based on the POD of reference method test portions using the Least Cost Formulations MPN calculator, with 95% confidence interval.

c N = Number of test portions.

d Results for candidate method presumptive, candidate method confirmed, and reference method were identical.

e x = Number of positive test portions.

f POD_CP_ = Candidate method presumptive positive outcomes divided by the total number of trials.

g POD_CC_ = Candidate method confirmed positive (per BAM Ch. 5) outcomes divided by the total number of trials.

h dPOD_CP_ = Difference between the candidate method presumptive result and candidate method confirmed result POD values.

i 95% CI = If the confidence interval of a dPOD does not contain zero, then the difference is statistically significant at the 5% level.

j Matrix tested by the independent laboratory.

k ATCC = American Type Culture Collection, Manassas, VA.

l N/A = Not applicable.

## Discussion

The results of this validation study show that the PhageDx *Salmonella* Assay is an effective alternative to the USDA/FSIS MLG 4.10 for the detection of *Salmonella* in 25 g raw ground turkey and FDA/BAM Chapter 5 for the detection of *Salmonella* in 100 g of milk-based PIF.

In inclusivity and exclusivity testing, the method was shown to be specific for *Salmonella*, correctly identifying 108 *Salmonella* target strains across both species and six *S. enterica* subspecies and 30 non-target strains. The PhageDx *Salmonella* Assay was unable to detect two strains within the inclusivity panel, a strain of *S. enterica, subsp. Arizonae* and of *S. enterica, subsp. Houtenae.* It is unclear as to why these strains were missed since the PhageDx *Salmonella* Assay was able to detect other members of the subspecies. One explanation is that these strains do not have the receptor(s) required for recognition by the phage. With over 2600 serovars in the genus, it is not surprising that there is a range of diversity that is difficult to encompass entirely. Another explanation may be that the strain has a mechanism that prevents the phage from replicating, thus unable to produce the luciferase reporter (12).

The recombinant phage can be produced consistently and is stable for 8 months when stored appropriately. Robustness testing of the PhageDx *Salmonella* Assay indicated that the method works well when the assay parameters (enrichment time, recombinant phage concentration, and substrate amount) were varied from the stated protocol.

Independent laboratory testing demonstrated that the PhageDx *Salmonella* Assay was able to detect *Salmonella* at low levels in 25 g test portions of raw ground turkey and 100 g test portions of milk-based PIF, which also contained approximately 3.6 × 10^6^ CFU/g and 1.8 × 10^3^ CFU/g background flora, respectively. These studies also demonstrated that the performance of the PhageDx *Salmonella* Assay was not statistically different from that of USDA/FSIS MLG 4.10 for 25 g test portions of raw ground turkey or FDA/BAM Chapter 5 for 100 g test portions of milk-based PIF. An alternative confirmation procedure was shown to be identical to the reference method confirmation procedures.

The PhageDx *Salmonella* Assay has a number of advantages over the USDA/FSIS MLG 4.10 and FDA/BAM Chapter 5 reference methods. In addition to being a specific assay, the results are easy to interpret as an RLU endpoint is used to determine the outcome of the assay. Another advantage is that PhageDx provides a presumptive positive result in as little as 9.5 h for raw ground turkey or 18.5 h for PIF compared to >24 h in the case of the USDA/FSIS MLG 4.10 and FDA/BAM Chapter 5 reference methods, respectively. PhageDx is also a simple test that involves only five basic steps: enrichment, sampling, infection, substrate addition, and signal readout. Finally, PhageDx Assay is a rapid method that offers a considerable time savings alternative compared to the USDA/FSIS MLG 4.10 and FDA/BAM Chapter 5 reference methods.

## Conclusion

Results of this validation study support the claim that the PhageDx *Salmonella* Assay is a specific, sensitive, fast, and simple method for the detection of *Salmonella* in raw ground turkey and milk-based PIF and is statistically comparable to the USDA/FSIS MLG 4.10 and FDA/BAM Chapter 5 methods, respectively. By using a luciferase-expressing recombinant bacteriophage, the assay was able to detect a single, viable bacterium after 7 h enrichment and a 2 h infection for raw ground turkey and 16 h enrichment and 2 h infection for milk-based PIF. The PhageDx *Salmonella* Assay thus offers shorter time to results compared with the other validated *Salmonella* detection assays.
